# Histamine Transmission Modulates the Phenotype of Murine Narcolepsy Caused by Orexin Neuron Deficiency

**DOI:** 10.1371/journal.pone.0140520

**Published:** 2015-10-16

**Authors:** Stefano Bastianini, Alessandro Silvani, Chiara Berteotti, Viviana Lo Martire, Gary Cohen, Hiroshi Ohtsu, Jian-Sheng Lin, Giovanna Zoccoli

**Affiliations:** 1 PRISM Laboratory, Department of Biomedical and Neuromotor Sciences, Alma Mater Studiorum University of Bologna, Bologna, Italy; 2 Department of Women & Child Health, Karolinska Institutet, Stockholm, Sweden; 3 Applied Quantum Medical Engineering, Graduate School of Engineering, Tohoku University, Sendai, Japan; 4 Physiologie intégrée du système d'éveil, Centre de recherche en neurosciences de Lyon, INSERM U1028-CNRS UMR 5292 Faculté de Médecine, Université Claude Bernard, Lyon, France; Hôpital du Sacré-Coeur de Montréal, CANADA

## Abstract

Narcolepsy type 1 is associated with loss of orexin neurons, sleep-wake derangements, cataplexy, and a wide spectrum of alterations in other physiological functions, including energy balance, cardiovascular, and respiratory control. It is unclear which narcolepsy signs are directly related to the lack of orexin neurons or are instead modulated by dysfunction of other neurotransmitter systems physiologically controlled by orexin neurons, such as the histamine system. To address this question, we tested whether some of narcolepsy signs would be detected in mice lacking histamine signaling (HDC-KO). Moreover, we studied double-mutant mice lacking both histamine signaling and orexin neurons (DM) to evaluate whether the absence of histamine signaling would modulate narcolepsy symptoms produced by orexin deficiency. Mice were instrumented with electrodes for recording the electroencephalogram and electromyogram and a telemetric arterial pressure transducer. Sleep attacks fragmenting wakefulness, cataplexy, excess rapid-eye-movement sleep (R) during the activity period, and enhanced increase of arterial pressure during R, which are hallmarks of narcolepsy in mice, did not occur in HDC-KO, whereas they were observed in DM mice. Thus, these narcolepsy signs are neither caused nor abrogated by the absence of histamine. Conversely, the lack of histamine produced obesity in HDC-KO and to a greater extent also in DM. Moreover, the regularity of breath duration during R was significantly increased in either HDC-KO or DM relative to that in congenic wild-type mice. Defects of histamine transmission may thus modulate the metabolic and respiratory phenotype of murine narcolepsy.

## Introduction

Narcolepsy is a chronic neurological disorder characterized by difficulty maintaining wakefulness, with daytime sleep lapses that often include rapid-eye-movement sleep (R) [1]. Narcolepsy type 1 is also characterized by cataplexy, which consists of transient loss of muscle tone during wakefulness, and by low cerebrospinal fluid (CSF) levels of the neuropeptide orexin A/hypocretin 1, which result from loss of hypothalamic orexin neurons [1, 2].

**Fig 1:**
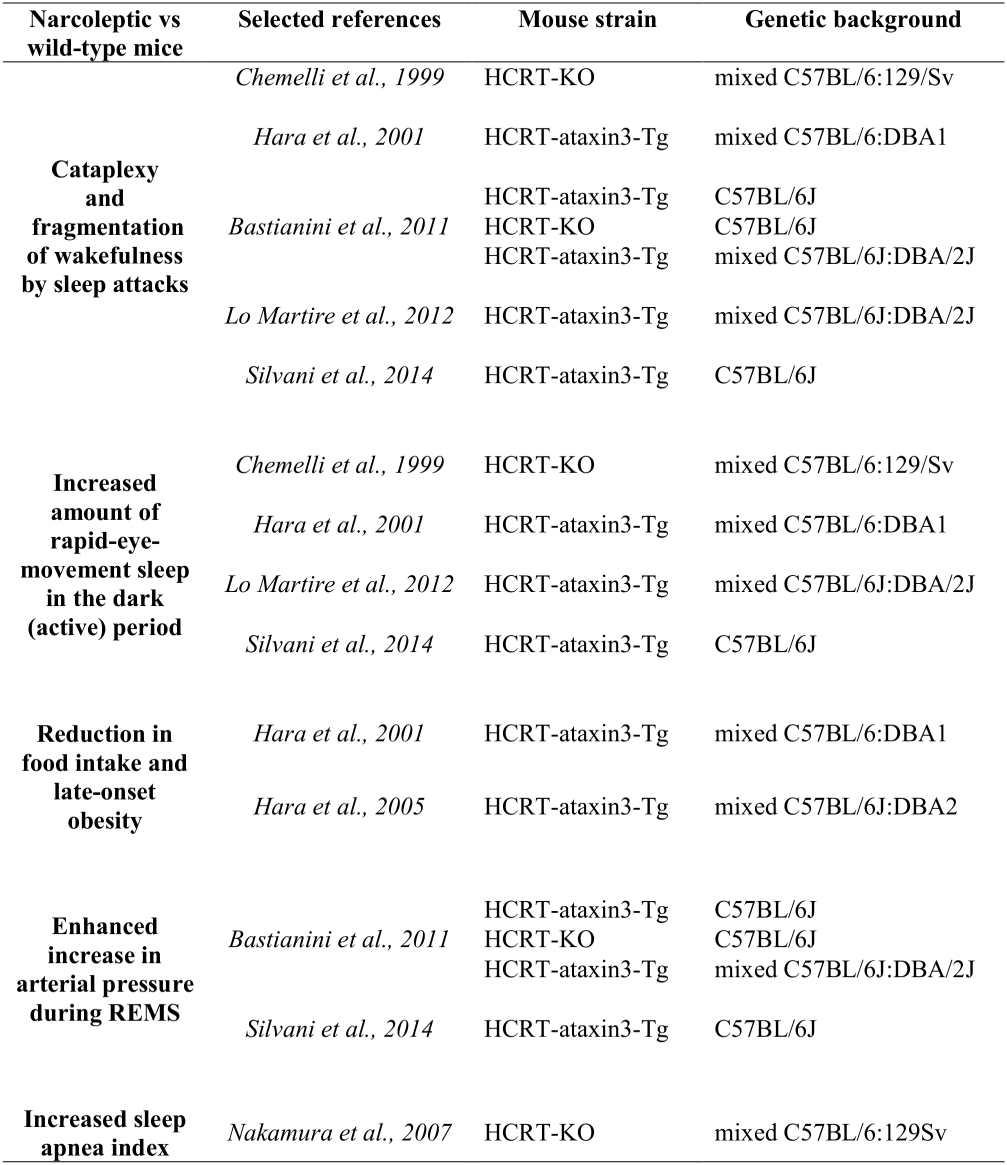
Narcolepsy characteristics described in different strains of male genetically-engineered orexin-deficient mice. HCRT-ataxin3-Tg: mice hemizygous for a transgene (hypocretin-ataxin3) coding for a neurotoxin, which causes selective ablation of orexin neurons; HCRT-KO: hypocretin gene knock-out mice with congenital deficiency of orexins.

A remarkable feature of narcolepsy type 1 is its wide comorbidity spectrum, which includes metabolic [3], cardiovascular [4], and respiratory [5] anomalies. These comorbidities also occur in orexin-neuron deficient mice (Fig 1), making a strong case for a causal role of orexin neuron loss. However, it is still unclear whether these different narcolepsy traits result from orexin neuron loss directly, because of the loss of direct orexinergic projections to neural structures involved in sleep, metabolic, and cardiorespiratory control, or rather indirectly, because of secondary and possibly compensatory imbalances of other transmitter systems. This complexity may contribute to changes of the narcoleptic phenotype among and within subjects, including peculiarities of childhood narcolepsy [6].

The hypothalamic histamine neurons are both excited [7, 8] and disinhibited [9] by orexin neurons. This strong functional link notwithstanding, published estimates of brain histamine levels in narcoleptic patients and orexin-deficient mice have been inconsistent, yielding either reduced [10, 11] or normal [12, 13] values compared to control subjects. Two independent studies even demonstrated an increased number of histamine neurons in the brains of narcoleptic patients at autopsy, perhaps as a compensation to their orexin deficit, but yielded contrasting results on orexin-deficient mice [14, 15]. These discrepancies may be partly explained by the technical challenges associated with histamine dosage and immunohistochemistry, and leave open the question of whether alterations of histamine signaling are associated to narcolepsy. Nonetheless, the enhancement of histamine transmission obtained by antagonism of H_3_ inhibitory histamine auto-receptors limits the excessive daytime sleepiness associated with narcolepsy [13, 16]. This finding indicates that imbalances of histamine transmission have the potential to impact significantly on narcolepsy pathophysiology, at least as far as the sleep phenotype is concerned. Histamine is also involved in metabolic [17], cardiovascular [18], and respiratory [19, 20] control. However, the potential for derangements of histamine transmission to modulate the metabolic, cardiovascular, and respiratory characteristics of narcolepsy has not been tested experimentally so far.

In order to address this question, we compared the phenotype of mice lacking the histamine-synthetizing enzyme (histidine decarboxylase; HDC-KO, [21]) and that of double mutant mice (DM) lacking histidine decarboxylase as well as orexin neurons due to the cell-specific expression of a neurotoxic transgene [22], with the phenotype of wild-type (WT) control mice with the same genetic background. We focused these comparisons on specific narcolepsy characteristics concerning sleep-wake derangements and cataplexy [1, 2], energy balance [3], cardiovascular control [4], and respiratory control [5]. These characteristics are relevant to human narcolepsy type 1, have been thoroughly described on orexin-deficient mouse models of narcolepsy, and have been already critically reviewed (cf., e.g., [23–25]). For ease of reference, the original reports on knock-out [26] and orexin-neuron ablated [22] mouse models, the first reports on the metabolic [27] and respiratory [28] characteristics of murine narcolepsy, and the research reports from our own laboratory concerning cardiovascular alterations in addition to cataplexy and sleep-wake characteristics of murine narcolepsy [29–31] are highlighted in Fig 1.

## Materials and Methods

### Ethics Statement

This study was carried out in accordance with the recommendations in the Guide for the Care and Use of Laboratory Animals of the National Institutes of Health. The protocol was approved by the Committees on the Ethics of Animal Experiments of the University of Bologna and of the Italian Ministry of Education, University, and Research (Permit Number: 8137). All surgery was performed under isoflurane anesthesia, and all efforts were made to minimize suffering.

### Mice

Experiments were performed on 11 HDC-KO mice knock-out for the histamine synthesis enzyme, L-histidine decarboxylase [21], and on 7 DM mice knock-out for L-histidine decarboxylase [21] as well as hemizygous carriers of the orexin-ataxin3 transgene, which causes postnatal loss of orexin neurons [22]. DM mice thus have orexin neuron deficiency, being a model of human narcolepsy type 1, and at the same time lack histamine. Founder mice carrying the orexin-ataxin3 transgene were provided generously by Prof. E. Mignot (Stanford University, Stanford, CA, USA). We compared HDC-KO and DM mice with 11 WT healthy control mice. All mice were adult (18–24 weeks at surgery) males and congenic to C57BL/6J (> 9 generations of back-crossing). The mouse colonies were maintained at the Department of Biomedical and Neuromotor Sciences of the University of Bologna, Italy. The homozygous HDC-KO mice under study resulted from homo- x homozygote and homo- x heterozygote breeding schemes. To obtain DM mice, we first bred HDC-KO homozygous mice with orexin-ataxin3 hemizygous mice. The progeny that resulted heterozygous for the HDC-KO mutation and hemizygous for the orexin-ataxin3 transgene was then crossed with HDC-KO homozygous mice to yield the DM mice under study, which were homozygous for the HDC-KO mutation and hemizygous for the orexin-ataxin transgene. WT mice were obtained with a WT x WT breeding scheme. In our animal facility, all founder lines are periodically backcrossed with C57BL/6J (Charles River, Calco, Italy), with less than 7 sibling (F) mating generations allowed between subsequent backcrosses. This periodically refreshes the genetic background avoiding the generation of substrains due to genetic drift.

Mice were housed under a 12:12-h light–dark cycle with ambient temperature set at 25°C and free access to water and food. Breeding pairs were fed standard rodent diet (4RF21 diet; Mucedola, Settimo Milanese, Italy). Experimental animals were weaned on the 4RF21 diet and switched thereafter to an open-source rodent diet (D12450B, Research Diets Inc., New Brunswick, NJ, USA; 3.85 Kcal/g; 20%, 70%, and 10% calories from proteins, carbohydrate, and fats, respectively), which guarantees full replicability of the dietary experimental conditions. This diet is free of histamine.

### Experimental Protocol

Surgery was performed under isoflurane anesthesia (1.8–2.4% in O_2_) with intra-operative analgesia (Carprofen 0.1 mg subcutaneously, Pfizer Italy, Latina) [32]. Mice were instrumented with electrodes for electroencephalography (EEG) and neck muscle electromyography (EMG) recordings. A calibrated telemetric arterial pressure transducer (TA11-PAC10, DSI, Tilburg, The Netherlands) was implanted subcutaneously, and the catheter tip was advanced via the femoral artery until it lay in the abdominal aorta below the renal arteries. After surgery, mice were housed individually and allowed 12–15 days to recover. Recordings were then made inside a whole-body plethysmograph to measure ventilation for 8 hours, starting at the onset of the light period (i.e., Zeitgeber Time 0, ZT0). After further 2–5 days’ recovery, mice underwent undisturbed 48 hours’ baseline recordings in their cages. Mice were then recorded while sleep-deprived by gentle handling for 6 hours (ZT0-ZT6) and during sleep recovery for 18 hours (ZT6-ZT24) [33].

### Data acquisition and analysis

The EEG, EMG and breathing of mice unrestrained except for the electrode tether were recorded continuously inside a modified 2-chamber whole-body plethysmograph (PLY4223, Buxco, Wilmington, NC, USA) [34]. The other recordings were performed on freely-behaving mice housed individually in cages with simultaneous acquisition of the EEG, EMG, and arterial pressure [32]. Data analysis was performed with MatLab (Mathworks, Natick, MA, USA). Scoring of wakefulness, non-rapid-eye-movement sleep (N), and R was performed visually by scrolling through raw EEG and EMG recordings at 4-s resolution [32]. The minimal duration of wakefulness, N, and R episodes was set at 12 s in sleep-wake structure analysis [29]. R episodes at sleep onset representing cataplexy-like events (CLE) in rodents were scored following consensus criteria [35]. The analysis was limited to CLE occurring during the dark (active) period of the day, which show a virtually complete specificity for cataplexy in mice [36]. Beat-to-beat mean arterial pressure and heart rate were computed from the raw arterial pressure signal in all artefact-free 4-s epochs [32]. Cardiovascular changes across state transitions were computed as previously described over the 48-hours’ baseline recordings [30]. Breathing analysis was performed on stable sleep-wake episodes lasting at least 12 s [34]. Individual breaths were identified automatically from the upward (+) plethysmograph pressure deflection peak. Errors in breath detection as well as pressure artefacts (e.g., due to movements) were manually excluded from the analyses. Stable, artefact-free periods of breathing comprised 71% and 68% of the N and R recordings, respectively, but only 5% of the time in wakefulness. A detailed analysis of breathing was therefore confined to periods of N and R. Instantaneous total breath duration, tidal volume, and minute volume (i.e., tidal volume divided by breath duration) were calculated, and volumes were expressed per gram body weight [34]. The variability of breath duration was analyzed with a technique originally proposed for the study of heart rate variability [37] and already applied to respiratory physiology [34, 38]. Briefly, the short-term (breath-to-breath) and long-term variability of breath duration were calculated based on Poincaré plots, in which the abscissa and ordinate of each point indicate the duration of the n^th^ and (n+1)^th^ successive breaths, respectively. In this analysis, the standard deviation of breath duration around the axis oriented with the line of identity of the Poincaré plot (SD_1_) estimates the short-term (breath-to-breath) variability of breath duration, while the standard deviation of breath duration around the orthogonal axis (SD_2_) estimates long-term variability [34, 37, 38]. The mean values of breath duration, tidal volume, and minute volume and the SD_1_ and SD_2_ of breath duration were computed for each mouse and sleep state after exclusion of the breaths with duration or and/ tidal volume that deviated more than 3 standard deviations from the respective mean value in the whole recording. These computations were thus protected from the effects of breaths with extreme values of duration and/or tidal volume. Finally, breaths with duration and tidal volume greater than 3 times average breath duration and tidal volume for each mouse in each sleep state were defined as breathing pauses (apneas) and augmented breaths (sighs), respectively, and each of them was visually checked on raw tracings to exclude artefacts [34].

### Statistics

Statistical tests were performed with SPSS (SPSS, Chicago, IL, USA) and significance at P < 0.05. Data are reported as means ± SEM. Differences in the distributions of the wakefulness episode duration were analyzed by Kolmogorov-Smirnov test. The other analyses were performed with ANOVA (GLM procedure with Huynh-Feldt correction when appropriate). Between-group differences were tested with t-tests only in case of significance of the main effect of group at ANOVA in order to limit type I errors.

## Results

### Cataplexy and sleep-wake derangements

**Fig 2:**
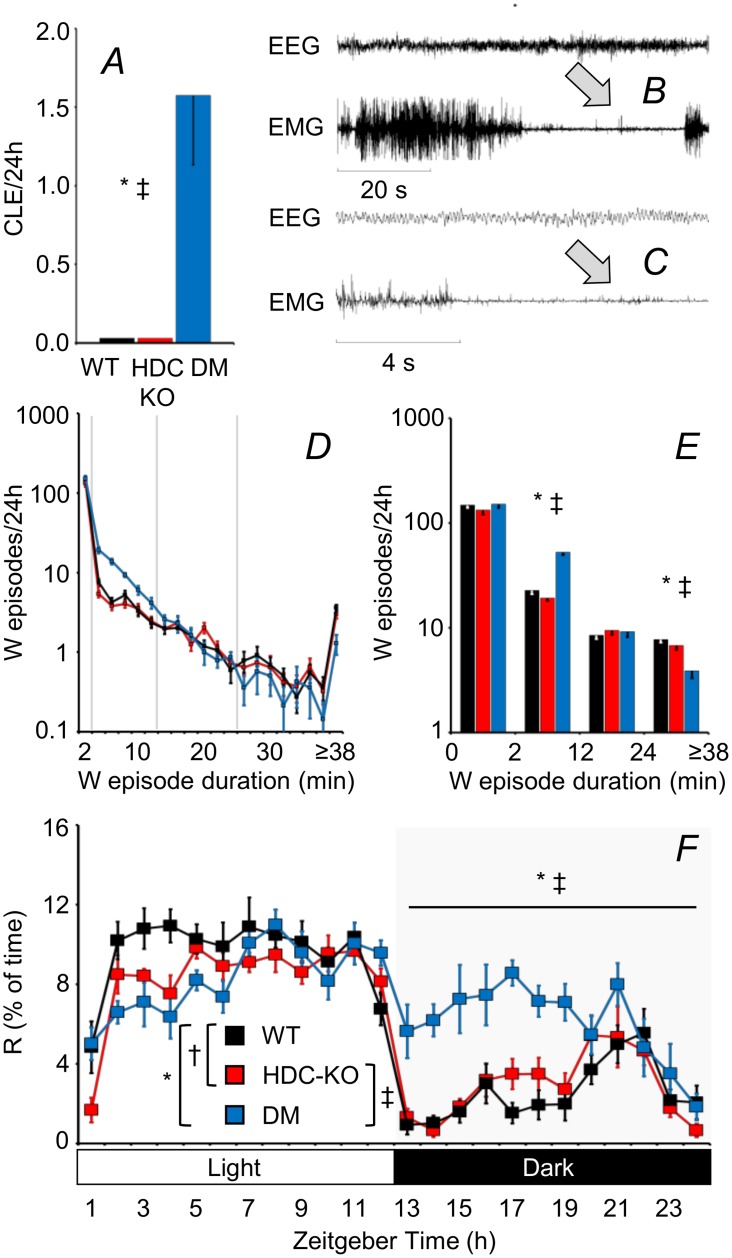
Cataplexy and sleep-wake derangements. (A) Frequency of occurrence of cataplexy-like episodes (CLE) during the dark period. In this and the remaining panels, data are means ± SEM in HDC-KO (n = 11), DM (n = 7), and WT (n = 11). (B) Representative raw tracings (EEG, electroencephalogram; EMG, electromyogram) showing a CLE (arrow) in a DM mouse. Details of the transition between wakefulness (W) and this CLE are shown in (C), evidencing an almost complete drop in neck muscle tone with preservation of the theta frequency rhythm of the preceding episode of W. (D) Frequency of occurrence of W episodes as a function of their duration. The distribution of W durations was significantly different between HDC-KO, DM, and WT (P < 0.001, Kolmogorov-Smirnov test). The bar graphs in (E) show the frequency of occurrence of W episodes in four arbitrary bins of W episode duration. (F) Percentage of recording time spent in rapid-eye-movement sleep (R). The horizontal bar refers to the whole dark period. *, †, and ‡, P < 0.05, WT vs. DM, WT vs. HDC-KO, and HDC-KO vs. DM, respectively (t-tests).

CLE during the dark period were detected in each DM mouse, but were absent in HDC-KO and WT (Fig 2A–2C). DM also showed fragmentation of wakefulness by sleep attacks (Fig 2D and 2E) and spent more time in R during the dark period (Fig 2F) compared to either WT or HDC-KO. No significant differences occurred between HDC-KO and WT. The percentage of time spent in wakefulness and non-rapid-eye-movement sleep (N) at baseline, and the responsiveness to a 6-hour sleep deprivation intervention in terms of sleep time and EEG slow-wave activity did not differ significantly among HDC-KO, DM, and WT (S1 and S2 Figs).

### Body weight and caloric intake

**Fig 3:**
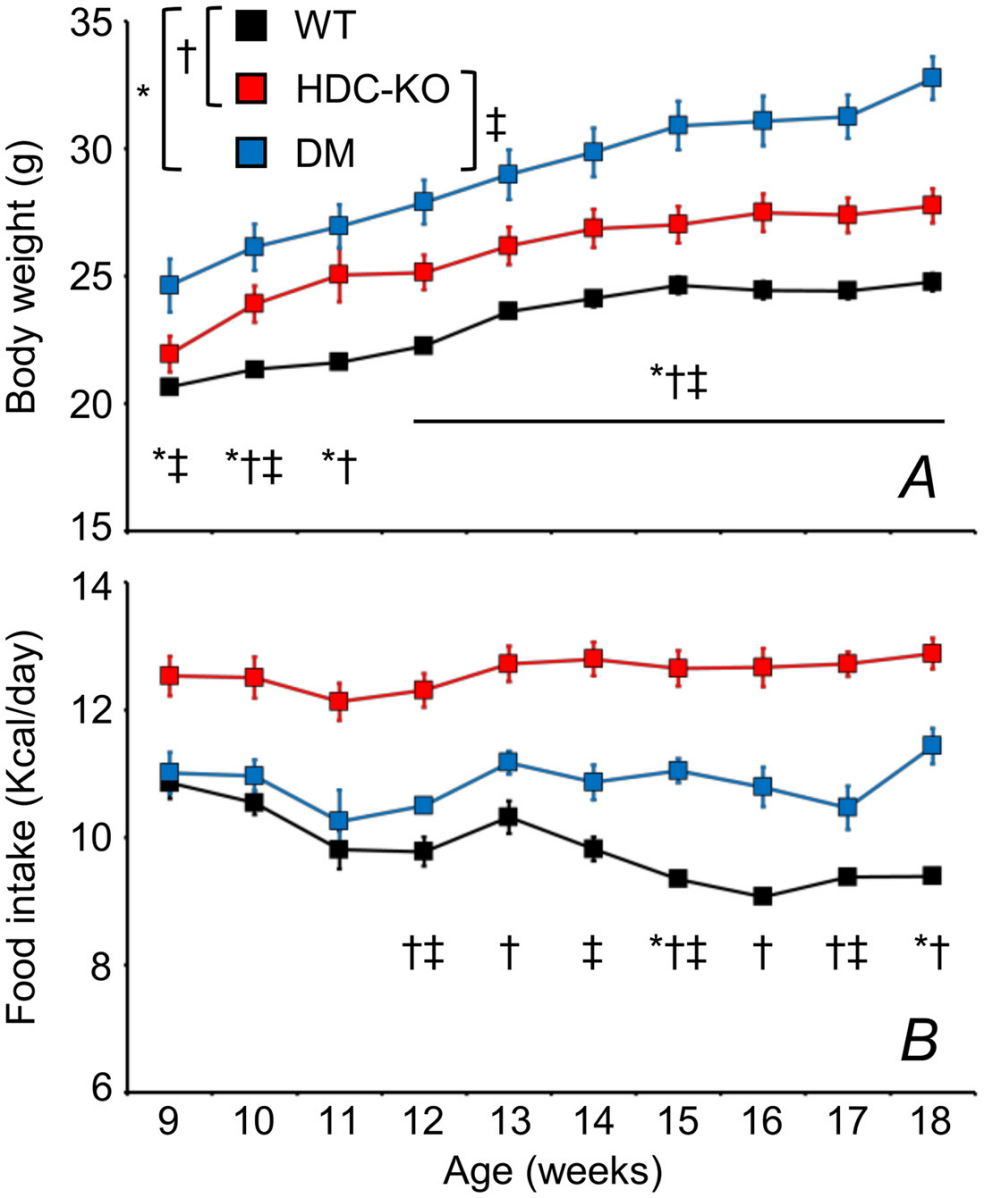
Body weight and caloric intake. Body weight (A) and nominal caloric food intake (B). Data are means ± SEM with n = 13–14 (WT), 10–14 (HDC-KO), and 3–8 (DM) at different time points. *, †, and ‡, P < 0.05, WT vs. DM, WT vs. HDC-KO, and HDC-KO vs. DM, respectively (t-tests). Symbols indicating significant differences above horizontal lines apply to each time point above the line.

Measurements of body weight and food consumption indicated that HDC-KO were heavier and ate more food than WT. DM showed a further increase in body weight compared not only with WT, but also with HDC-KO, but their caloric intake was intermediate between those of HDC-KO and WT (Fig 3).

### Cardiovascular regulation

**Fig 4:**
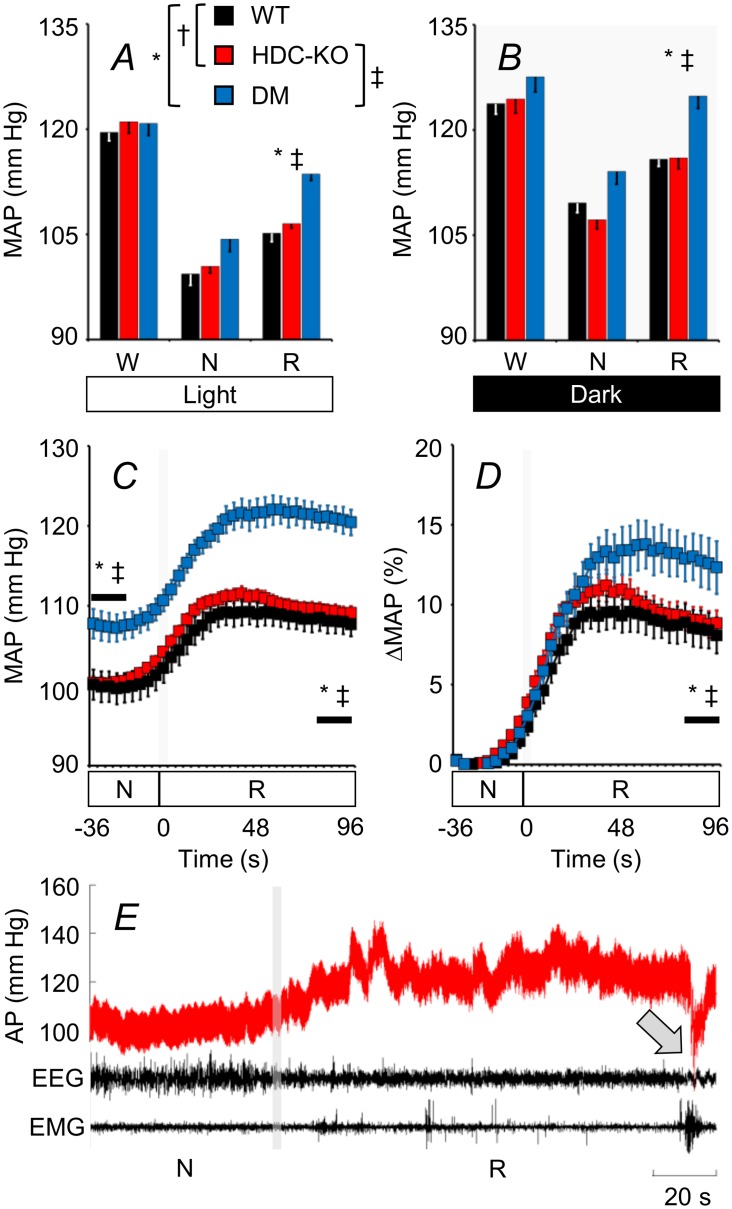
Sleep-related changes in arterial pressure. (A) and (B), values of mean arterial pressure (MAP) during wakefulness (W), non-rapid-eye-movement sleep (N), and rapid-eye-movement sleep (R) in the light and dark periods, respectively. In this and the other panels, data are means ± SEM in HDC-KO (n = 11), DM (n = 7), and WT (n = 11). (C) and (D), time course of MAP changes during transitions between N and R shown in absolute values and in percentage of the values at baseline, respectively. Horizontal bars refer to the first and the last 20 s of the transitions. E, representative tracing of MAP, electroencephalogram (EEG) and electromyogram (EMG) during a transition from N to R in a DM mouse, highlighting a dramatic and sustained arterial pressure (AP) increase in R. The arrow in E indicates the awakening from R. The grey vertical bars in C, D, and E indicate the transition point between states. *, †, and ‡, P < 0.05, WT vs. DM, WT vs. HDC-KO, and HDC-KO vs. DM, respectively (t-tests).

DM had higher arterial pressure during R than either HDC-KO or WT in the light and dark periods (Fig 4A and 4B). Analysis of transitions from N to R revealed that arterial pressure was also higher in DM than in either HDC-KO or WT during the last part of the N episodes before the transitions to R (Fig 4C), and increased more in DM than in either HDC-KO or WT on passing from N to R (Fig 4D). No significant differences occurred between HDC-KO and WT.

### Respiratory regulation

**Fig 5:**
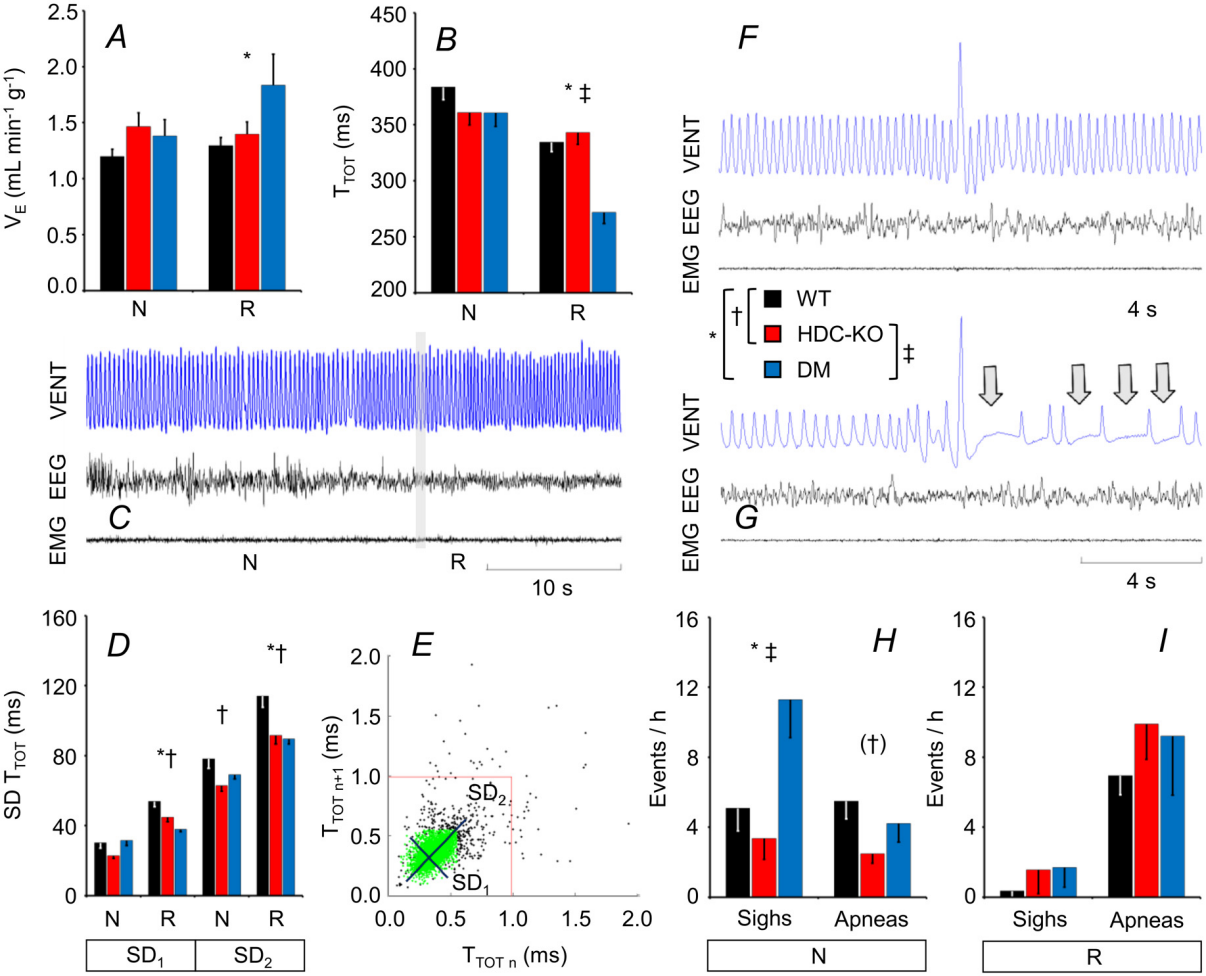
Sleep-related changes in breathing. (A) and (B), values of minute volume (V_E_) and breath duration (T_TOT_), respectively, during non-rapid-eye-movement sleep (N) and rapid-eye-movement sleep (R). In this and the other panels, data are means ± SEM in HDC-KO (n = 11 during N, n = 10 during R), DM (n = 7), and WT (n = 11). (C) Representative tracing (plethysmographic ventilator signal, VENT; electroencephalogram, EEG; electromyogram, EMG) during a transition from N to R in a DM mouse. The decrease in T_TOT_ during R is evident from the occurrence of more closely spaced deflections of the VENT signal with individual breaths. The grey vertical bar shows the transition point between states. (D) Short-term (SD_1_) and long-term (SD_2_) variability of T_TOT_. (E) Representative Poincaré plot during N in a DM mouse, in which abscissa and ordinate of each point indicate T_TOT_ of successive breaths. SD_1_ and SD_2_ correspond to the standard deviations around axes (black segments) oriented with or orthogonal to the line of identity of the Poincaré plot, respectively. SD_1_ and SD_2_ are computed excluding extreme values of T_TOT_ (black points). The red lines mark the threshold for apnea detection (i.e., three times the average T_TOT_ value). (F) and (G), representative tracings during N in a DM mouse showing augmented breaths (sighs) either isolated (F) or followed by breathing pauses (apneas; G, arrows). (H) and (I), frequency of occurrence of sighs and apneas during N and R, respectively. *, †, and ‡, P < 0.05, WT vs. DM, WT vs. HDC-KO, and HDC-KO vs. DM, respectively (t-tests). In panel H, the symbol (†) indicates a statistical tendency for the difference between HDC-KO and WT (P = 0.051, ANOVA; HDC-KO vs. WT, P = 0.017, t-test).

Neither mean minute volume nor mean breath duration during sleep differed significantly between HDC-KO and WT. Conversely, DM had significantly greater minute volume during R than WT. There was a statistical tendency (P = 0.054) for this difference to be significant also between DM and HDC-KO (Fig 5A). This occurred because of a significant reduction in breath duration during R in DM compared with either HDC-KO or WT (Fig 5B and 5C), whereas tidal volume did not differ (S3 Fig). HDC-KO had significantly lower short-term and long-term variability of breath duration during R and significantly lower long-term variability of breath duration during N than WT. Similar differences occurred during R in DM compared with WT (Fig 5D and 5E). Augmented breaths (sighs) during N were significantly more frequent in DM than either in HDC-KO or WT, whereas their occurrence rate did not differ significantly between HDC-KO and WT. The apnea index in N tended to be lower in HDC-KO than in WT (P = 0.051, ANOVA; HDC-KO vs. WT, P = 0.017, t-test), whereas it did not differ significantly between DM and WT. HDC-KO, DM, and WT did not differ significantly in terms of sighs and apneas in R (Fig 5H and 5I).

## Discussion

Our study yielded different novel findings. In particular, we found that cataplexy, fragmentation of wakefulness by sleep attacks, an increased amount of R sleep in the dark (active) period, and an enhanced increase in arterial pressure during R occurred in DM but not in HDC-KO mice compared with WT. The occurrence of these phenotypic characteristics, which are hallmarks of murine narcolepsy, therefore does not require histamine transmission and is not caused by histamine deficiency itself. Conversely, increases in body weight and food consumption and increases in the stability of breath duration during sleep occurred both in HDC-KO and in DM compared with WT. This indicates a potential for histamine deficiency to modulate the metabolic and respiratory phenotype of narcolepsy.

Cataplexy and fragmentation of wakefulness by sleep attacks are defining features of narcolepsy type 1 in human patients [1], and have been robustly reported in orexin-deficient narcoleptic mice (Fig 1). Increased propensity to R is another characteristic feature of narcoleptic patients [1, 39], which manifests dramatically also in orexin-neuron deficient mice (Fig 1), determining a deficit of the circadian suppression of R during the dark (active) period of the day [40]. We found that cataplexy, fragmentation of wakefulness by sleep attacks, and excess R during the dark (active) period did not occur in HDC-KO, but only in DM (Fig 2). Our findings indicate that the occurrence of these narcoleptic signs does not require the integrity of histamine transmission and is not caused by histamine deficiency by itself. We also demonstrated for the first time that histamine is not required for normal responsiveness to a 6-hour sleep deprivation intervention in terms of sleep time and EEG slow-wave activity (S2 Fig,). Therefore, just as in narcoleptic patients [41, 42] and orexin knock-out mice [43], the fragmentation of wakefulness and the excess R sleep time in the dark period, which we found in DM compared with WT, were not produced by an enhanced homeostatic buildup of N and/or R need during wakefulness. On the other hand, our finding that the amount and distribution of wake-sleep states in the light and dark periods did not differ significantly between HDC-KO and WT (Fig 2; S1 and S2 Figs) is in accordance with data on histamine H1 receptor KO mice [44]. However, these findings are at variance with the known acute hypnotic effect of H_1_ histamine receptor blockers [17] and with a previous report of fragmentation of wakefulness and reduced time spent in wakefulness at lights off in HDC-KO mice on the 129/Sv background [45, 46]. These discrepancies raise the hypotheses that the effects of the lack of histamine on the 24-h sleep-wake profile can be compensated in the long run to an extent, which is greater on the C57BL6/J background than on the 129/Sv background.

Orexins increase food intake and energy expenditure [47]. Accordingly, reductions in food intake and energy expenditure have been reported in orexin-deficient narcoleptic patients [3, 48, 49] and orexin-neuron deficient mice [50]. Although reductions in food intake and energy expenditure have opposing effects on energy balance that may compensate, the reduction in energy expenditure tends to prevail in narcoleptic patients. Accordingly, obesity is frequent in narcoleptic patients [3, 48, 49], particularly in children shortly after disease onset [6, 51]. Conversely, obesity occurs in orexin-deficient mouse models of narcolepsy only late in life (Fig 1) if at all, and appears modulated by age, sex, and orexin co-transmitters [27, 52]. In particular, orexin-neuron deficient male mice do not show obesity at least up to 43 weeks of age on the C57BL/6J background [27, 29, 31, 52], whereas they develop obesity at 14–16 weeks of age on mixed C57BL/6-DBA backgrounds [22, 27, 52]. We found that male DM on the C57BL/6J background were obese and ate more than WT (Fig 3). Body weight of male HDC-KO on the same genetic background was intermediate between those of WT and DM, whereas food intake was the highest of the three groups. These data agree with the known anorexigenic action of histamine [17], with a previous report of increased body weight in HDC-KO mice on the 129/Sv background [46], and with preliminary data on double-knock-out mice for HDC and orexin [53]. This indicates that effects of histamine on energy balance are more robust than those on sleep-wake control to changes in genetic background. Our results also indicate that DM were obese because of increased energy intake coupled with reduced energy expenditure, at least compared with HDC-KO. Together, these results demonstrate a potential for defects in histamine transmission to counteract the reduction in food intake caused by orexin neuron deficiency, and thereby promote obesity in presence of reduced energy expenditure. Other hypothalamic transmitters modulate energy balance in addition to histamine [54], but their potential to modulate the narcoleptic phenotype has not been tested so far. Our results raise the hypotheses that CSF histamine levels are reduced in obese narcoleptic children and correlate negatively with body mass index in adult narcoleptic patients.

Both in orexin-deficient humans [4, 55, 56] and mice (Fig 1), narcolepsy entails a reduced difference in arterial pressure between wakefulness and sleep, which is most evident in R. This state represents a significant fraction of total sleep time in healthy subjects as well as in narcoleptic patients (20%-25%, cf. [4, 55]). This is of interest because increases in arterial pressure during sleep (non-dipping arterial pressure pattern) are known to increase cardiovascular risk significantly [57]. On the other hand, cardiovascular and renal pathology in middle-aged narcoleptic mice does not show any obvious alteration compatible with chronic arterial pressure elevation [31], and adverse effects associated to increased sleep arterial pressure in narcoleptic patients may be offset by reduced sympathetic activity during wakefulness [58]. Thus, it is still an open question whether the enhanced increase in arterial pressure during R contributes to the increased cardiac comorbidity [59] and mortality rate [60], which have recently been reported for narcoleptic patients. We found that arterial pressure during R and during N immediately before R was significantly higher in DM than either in HDC-KO or WT, whereas it did not differ significantly between HDC-KO and WT (Fig 4). Thus, the occurrence of inappropriately high values of arterial pressure during R did not require the integrity of histamine transmission and was not caused by histamine deficiency by itself. Recent evidence indicates that histamine raises arterial pressure by acting on the nucleus of the solitary tract [18], which is thought to play a key role in sleep-dependent cardiovascular control [61]. In this light, our finding that arterial pressure did not differ significantly between HDC-KO and WT as a function of the wake-sleep state (Fig 4) is also of physiological interest because it indicates that histamine modulation of the nucleus of the solitary tract is not critical for the occurrence of physiological sleep-related changes in arterial pressure.

Sleep-disordered breathing is highly prevalent in narcoleptic patients [62–64] and may prominently involve central apneas [5]. Accordingly, the incidence of central apneas is higher in orexin knock-out mice than in WT both during N and during R (Fig 1). Moreover, post-hypoxic long-term facilitation, a physiological response that is presumed to stabilize the respiratory control system and reduce sleep apnea, is absent in orexin knock-out mice [65]. By contrast, we found that DM did not have any increase in sleep apnea index compared with WT, but rather had lower breath-to-breath (SD1) and long-term (SD2) variability of breath duration during R compared with WT (Fig 5). HDC-KO also had decreased variability of breath duration during sleep and tended to have a lower apnea index during N compared with WT (Fig 5). The finding of a similar reduction in the variability of breath duration during R in HDC-KO and DM compared with WT indicates a potential for histamine deficiency to modulate the respiratory phenotype of narcolepsy, at least during R, in the sense of a greater regularity of breathing. Further studies are needed to evaluate whether and to what extent this effect impacts on blood-lung gas exchange during sleep and sleep stability. Further work will also be needed to clarify whether the combined lack of orexin and histamine explains the increase in minute volume during R and the increase in sigh rate during N, which we found in DM compared with either HDC-KO or WT (Fig 5), and which have not been previously observed on orexin-deficient mice [28]. Taken together with the enhanced increased in arterial pressure during R (Fig 4), the increased minute volume during R (Fig 5) indicates a powerful and combined somatic and autonomic neural activation in DM. In light of the reported changes in histamine neurons in narcolepsy [14, 15], this opens ways to speculation about a role of non-histamine-dependent actions of histamine neurons, such as GABAergic neurotransmission [17], in respiratory control. On the other hand, both orexins [66] and histamine [67] modulate neurons of the medullary pre-Bötzinger complex, which is strongly implicated in the genesis of sighs [68]. Finally, histamine is known to affect breathing pattern formation in the brainstem respiratory network acting on H_1_ receptors [19, 20]. Our findings demonstrate for the first time that histamine deficiency entails respiratory consequences during sleep, which is remarkable because histamine neurons are active only during wakefulness [69]. A similar paradox occurs with orexin neurons, which are also wake-active [70], and whose lack causes several phenotypic alterations during sleep in narcolepsy (Fig 1). In either case, sleep effects may result from the loss of residual brain interstitial concentration of orexins/histamine or from carryover during sleep of mechanisms, which compensate for loss of orexin/histamine effects during wakefulness (cf. [29]).

A limitation of our study is that we did not perform experiments on littermate mice. While this limitation is lessened because we carefully avoided generating genetic substrains (cf. Methods), it did not allow us to control, e.g., for possible long-term effects of differences in parental care between genotypes. On the other hand, this design allowed us to employ breeding strategies that greatly reduced the generation of unusable mice with genotypes different from those of interest, in line with the “3r” ethical principles (cf., e.g., http://www.basel-declaration.org/). Another limitation of our study is that we did not perform parallel 4-genotype experiments including mouse carriers of the orexin-ataxin3 transgene without the HDC-KO mutation. As the specific narcolepsy characteristics under study have already been thoroughly described on orexin-deficient mouse models of narcolepsy (Fig 1), this was aimed to avoid potentially unnecessary duplication of experiments, again in line with the “3r” reduction principle. However, we acknowledge that our present results are not amenable to direct quantitative and statistical comparison with those listed in the Fig 1 because they were not obtained in parallel with the same experimental protocol. Our experimental design thus prevented us to infer whether the magnitude of phenotypic differences associated with orexin neuron loss, such as those in sleep-wake behavior, cataplexy, and arterial pressure, was modified by the concomitant lack of histamine. However, our experimental design did allow us to investigate for the first time the effects of histamine deficiency on the occurrence (yes/no, in terms of presence or absence of significant differences in the expected direction compared with WT) of specific narcolepsy traits.

## Conclusions

We found that the occurrence of cataplexy, fragmentation of wakefulness by sleep attacks, increased amount of R sleep in the dark (active) period, and enhanced increase in arterial pressure during R, which are hallmarks of murine narcolepsy, do not require histamine transmission and are not caused by its deficiency. Conversely, our results indicate a potential for histamine deficiency to modulate the metabolic and respiratory phenotype of narcolepsy by increasing food intake, body weight, and the regularity of breathing during sleep.

## Supporting Information

S1 Fig Percentage of the recording time spent in wakefulness (W, panel A) and non-rapid-eye-movement sleep (N, panel B) with mice undisturbed in the home cage. Data are means ± SEM in HDC-KO (n = 11), DM (n = 7), and WT (n = 11).
(TIF)

S2 Fig Assessment of the responsiveness to a 6-hour sleep deprivation intervention. (A) Increase in electroencephalographic slow-wave activity (SWA) during non-rapid-eye-movement sleep (N) in the recovery period after sleep deprivation, expressed as percentage of the values in the last 4 hours of the light period at baseline. In this and the other panels, data are means ± SEM in HDC-KO (n = 11), DM (n = 7), and WT (n = 11). (B) Percentage of N and rapid-eye-movement sleep (R) time lost during sleep deprivation, respectively, which was recovered at the end of the sleep recovery period. (C) and (D), percentage of recording time spent in N and R, respectively, during sleep deprivation and recovery. The percentage of recording time spent in R during the dark period was significantly higher in DM than either in HDC-KO or WT after sleep deprivation, similarly to what occurred at baseline before sleep deprivation (D, horizontal bar). * and ‡, P < 0.05, WT vs. DM and HDC-KO vs. DM, respectively (t-tests).
(TIF)

S3 Fig Values of tidal volume (V_T_) during non-rapid-eye-movement sleep (N) and rapid-eye-movement sleep (R). Data are means ± SEM in HDC-KO (n = 11 in N, n = 10 in R), DM (n = 7), and WT (n = 11).
(TIF)

S1 File Original data and related metadata underlying the findings reported in the submitted manuscript. (XLSX)
