# Supplementary figures and images for: Histamine Transmission Modulates the Phenotype of Murine Narcolepsy Caused by Orexin Neuron Deficiency
Source: PLoS One. 2015 Oct 16;10(10):e0140520. doi: 10.1371/journal.pone.0140520 (PMC4608736; doi:10.1371/journal.pone.0140520)

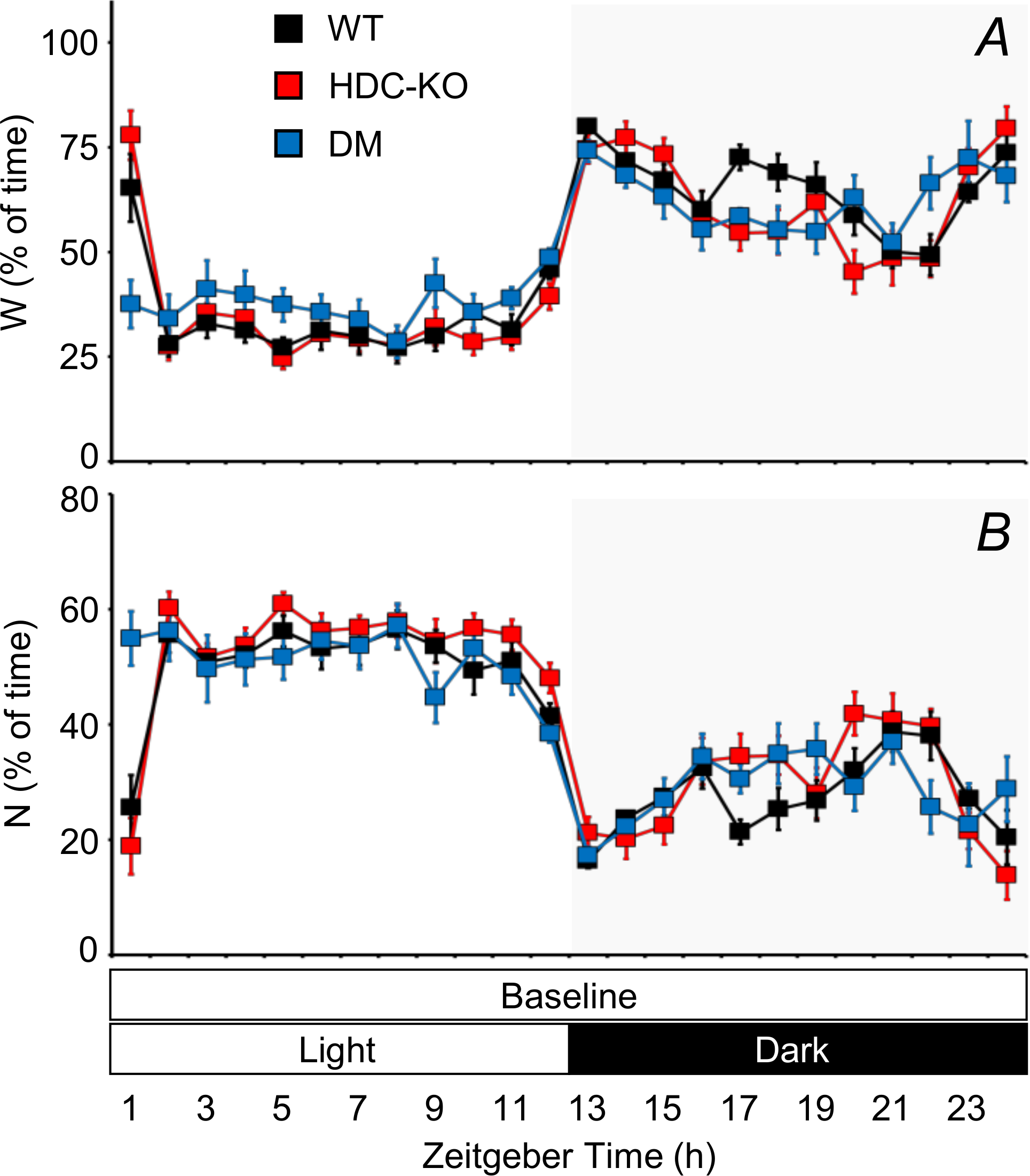

Supplement: S1 Fig — Data are means ± SEM in HDC-KO (n = 11), DM (n = 7), and WT (n = 11). (TIF) [file pone.0140520.s001.tif]

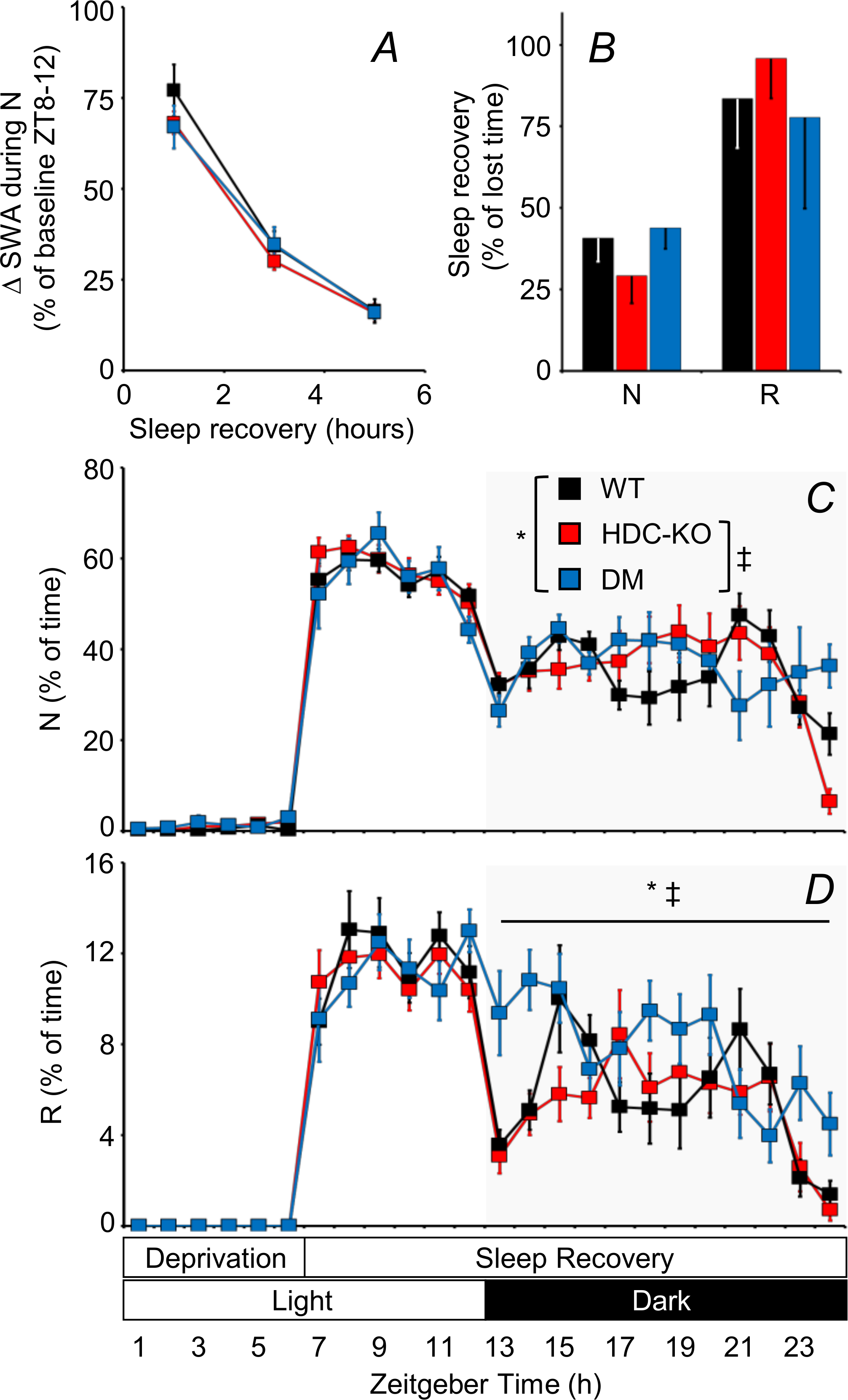

Supplement: S2 Fig — (A) Increase in electroencephalographic slow-wave activity (SWA) during non-rapid-eye-movement sleep (N) in the recovery period after sleep deprivation, expressed as percentage of the values in the last 4 hours of the light period at baseline. In this and the other panels, data are means ± SEM in HDC-KO (n = 11), DM (n = 7), and WT (n = 11). (B) Percentage of N and rapid-eye-movement sleep (R) time lost during sleep deprivation, respectively, which was recovered at the end of the sleep recovery period. (C) and (D), percentage of recording time spent in N and R, respectively, during sleep deprivation and recovery. The percentage of recording time spent in R during the dark period was significantly higher in DM than either in HDC-KO or WT after sleep deprivation, similarly to what occurred at baseline before sleep deprivation (D, horizontal bar). * and ‡, P < 0.05, WT vs. DM and HDC-KO vs. DM, respectively (t-tests). (TIF) [file pone.0140520.s002.tif]

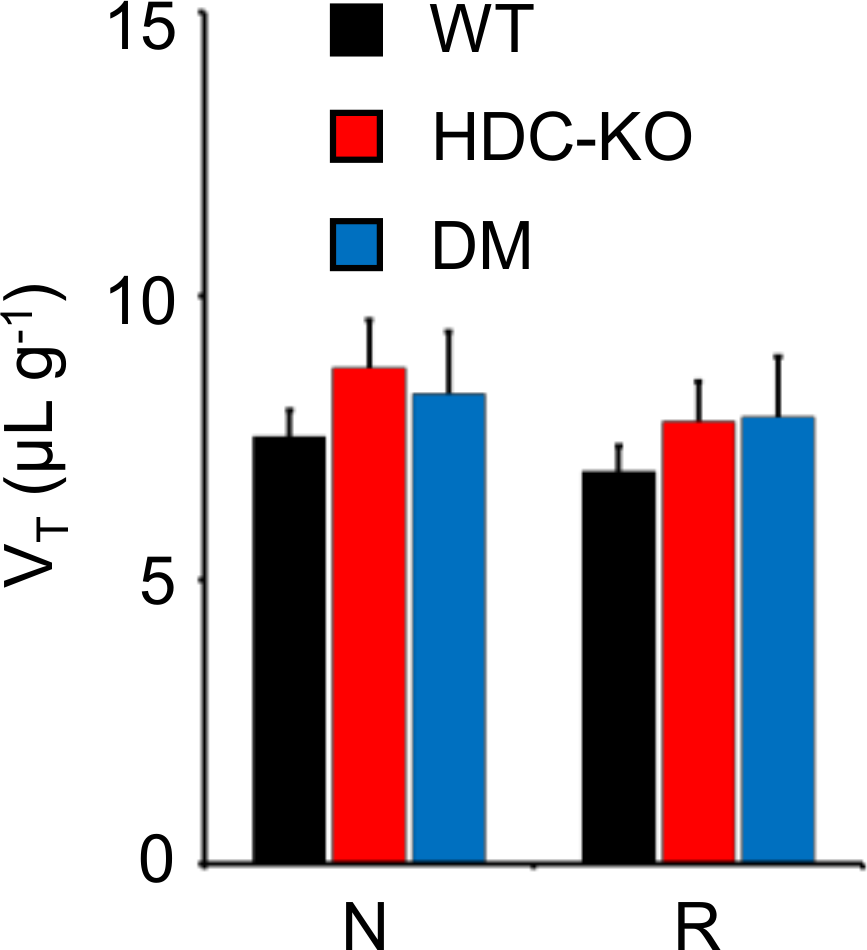

Supplement: S3 Fig — Data are means ± SEM in HDC-KO (n = 11 in N, n = 10 in R), DM (n = 7), and WT (n = 11). (TIF) [file pone.0140520.s003.tif]
